# Editorial: The evolution of minimally invasive urologic surgery: innovations, challenges, and opportunities

**DOI:** 10.3389/fsurg.2024.1525713

**Published:** 2024-12-11

**Authors:** Lazaros Tzelves, Patrick Juliebø-Jones, Bhaskar Somani

**Affiliations:** ^1^ 2nd Department of Urology, Sismanoglio Hospital, National and Kapodistrian University of Athens, Athens, Greece; ^2^ Department of Urology, Haukeland University, Bergen, Norway; ^3^ Department of Urology, University of Southampton, Southampton, United Kingdom

**Keywords:** robotic surgery, urology, endourology, suction, artificial intelligence, chatGPT, kidney calculi, ureteroscopy

**Editorial on the Research Topic**
The evolution of minimally invasive urologic surgery: innovations, challenges, and opportunities

Technological advances have had a great impact on the evolution of medicine and contributed to improvements in surgical technique. In this regard, the field of urology is no exception. Over the past decade, several technological innovations have led to new challenges and opportunities both in terms of diagnosing and treating benign and malignant urological conditions.

Large language models (LLMs) represent a rapidly expanding field, with many tested applications in the urological setting already. Talyshinskii et al. provide an insightful summary of one of the most widely used LLMs, ChatGPT. The authors emphasize its role in drafting clinical documents and notes, in facilitating communication with patients, while medical students and clinicians can benefit from an educational and research perspective Talyshinskii et al. of course, attention is needed to avoid use of misleading or even fake references, plagiarism, scientific fraud, issues with patients’ data privacy and isolation between patient and physician Talyshinskii et al.

Social media (SoMe) is a means of expression, while it also represents a way to communicate concerns, experiences and perceptions, especially regarding several health issues. Juliebø-Jones et al. evaluated one of the most popular SoMe platforms, TikTok, regarding kidney stone surgery. The authors included the 100 most recent video posts and found that that the majority of posts were about recovery, pain and stents, while 51% showed a negative tone Juliebø-Jones et al.

Prostate cancer is one of the most common cancers in men, therefore it is reasonable that a lot of research focuses on this field. Haack et al. designed a comparative study to assess the ability of urologists to localize suspicious cancer lesions on multi-parametric (mp) magnetic resonance imaging (MRI), when having only mpMRI images, mpMRI images with radiological reports and mpMRI images with 3D printed model; they reported that radiology reports are still needed, while 3D models seem to be efficient, especially in younger residents Haack et al. Radical prostatectomy still represents a main form of management of localized disease and while it offers high survival rates, two of its associated sequelae, incontinence and erectile dysfunction, hinder its popularity. Leitsmann et al. assessed the impact of mpMRI-targeted biopsy on functional outcomes in patients undergoing robot-assisted radical prostatectomy Leitsmann et al. They reported that mpMRI-targeted biopsy compared to standard biopsy, led to fewer positive surgical margins, lower risk of erectile dysfunction at 1-year, lower rate of postoperative tumor upgrading and, in cases of nerve-sparing approach, fewer secondary nerve resection Leitsmann et al. Katsimperis et al. provided a concise summary with their narrative review on the approaches used to preserve continence after robot-assisted radical prostatectomy; bladder neck preservation, neurovascular bundle preservation, preservation of apical intraprostatic urethra, Retzius-sparing and hood techniques, anterior and/or posterior reconstructive stiches and newer techniques such as complete urethral preservation (CUP) and single port transvesical robotic prostatectomy Katsimperis et al. A critical step of radical prostatectomy, where a large amount of blood loss can occur, is deep vascular complex ligation. Chen et al. described a simulation platform for training of novice surgeons and residents on this step, showing good construct, face and content validity, while maintaining low costs Chen et al.

Partial nephrectomy is the treatment of choice in cases of T1 renal tumors and in selected cases in larger neoplasms, in order to maximize renal function preservation. Conventional technique, either performed via an open or minimally invasive approach, consists of clamping the renal arterial supply (warm ischemia) and subsequently excising the tumor and suturing the tissue defect, usually in two layers (inner and outer renorrhaphy). Several techniques have been described, with the sliding technique using clips to support the tissues and barbed sutures being the most commonly used one. Nguyen et al. performed an *in vivo* study to evaluate the use of off-clamp, microwave scissors-based and sutureless partial nephrectomy technique, compared to on-clamp conventional approach; they reported that the former one exhibited reduced surgical time and less normal nephron loss, while blood loss and urinoma formation were not significantly different Nguyen et al. Tumor characteristics play a major role in surgical complexity, while it also drives the complication profile in every patient. Gu et al. summarized existing evidence in their systematic review and meta-analysis regarding impact of completely endophytic renal masses Gu et al. They calculated based on six studies involving 2,126 patients that completely endophytic tumors compared to non-endophytic exhibit significantly higher rates of major complications, longer warm ischemia time, greater drop in renal function and lower rates of trifecta achievement Gu et al.

Urolithiasis in the upper urinary tract is a very common benign clinical condition affecting nearly 10% of population. Several advances have been performed in this field; Juliebø-Jones et al. provide an overview of controversies in endourology by evaluating the role of single use ureteroscopes and optimal use of laser for lithotripsy, comparing basketing vs. dusting techniques, assessing the impact of ureteral access sheath and the necessity of safety guidewires and finally providing a balanced conclusion for readers Juliebø-Jones et al. Achieving stone-free status (SFS) with minimal complications and reduced operative times are the main primary outcomes in endourological treatment of urolithiasis, thus also in ureteroscopy. During the last years we have seen an uprise in the use of suction via access sheaths, ureteroscopes or nephroscopes; use of suction achieves two main goals: minimizes intrarenal pressure and aids in removal of small fragments, thus avoiding repetitive extraction of fragments with baskets or forceps. Reduced intrarenal pressure minimizes complications by avoiding pyelovenous and pyelolymphatic backflow of urine and microorganisms. Zhang et al. evaluated a suctioning ureteral access sheath for removal of upper tract stones under local anaesthesia Zhang et al. In their study, authors described a feasible operating under local anaesthesia for a mean stone size of more than 2 cm and final SFS equal to 85.1% Zhang et al., thus showing clinical effectiveness of this technique. Endourological techniques have been applied also in cases of upper urinary tract urothelial carcinoma. Chen et al. assessed the clinical efficacy of an intelligent-pressure controlled ureteroscope with Thulium laser fiber (TFL) in treating patients with isolated upper urinary tract urothelial carcinoma Chen et al. This study focused on six patients, whose surgeries were smooth with no intraoperative complication, thus indicating that this technique might be feasible for this purpose.

Stress urinary incontinence in men can be observed mainly after treatments for prostate cancer, i.e., radical prostatectomy or radiation therapy and can lead to serious compromise of quality of life. In moderate-severe stress incontinence, surgical management is indicated, with male slings being one of the available choices. Adjustable transobturator male system (ATOMS^TM^, A.M.I., Austria) is a treatment option for which a growing body of evidence exists. Juliebø-Jones et al. in their narrative review provide an updated summary on relevant evidence, showing that ATOMS may offer effectiveness similar to artificial urinary sphincter, while it provides the opportunity to replace certain parts of the device without replacing the device itself Juliebø-Jones et al.

Finally, several interesting case reports and case series are presented in this special issue. Yao et al. described their experience with an extrarenal renal cell carcinoma (RCC) in the adrenal region; a 48-year old lady and an isolated adrenal tumor had surgery, which revealed a clear-cell carcinoma, reminding us that RCC belongs to the differential diagnosis of adrenal masses Yao et al. Staying on the same subject of adrenal masses, Shi et al. described a solitary fibrous tumor of the adrenal gland, emphasizing the importance of this differential diagnosis in patients with low-density and uneven CT enhancement features Shi et al. Huang et al. published their technique of minimally-invasive single-port laparoscopic repair of vesicovaginal fistula through the vagina of a 53-year old female patient; this description represents the first “zero incision” technique for single-port laparoscopy in patients with high-position vesico-vaginal fistula and is accompanied by educational and explanatory figures Huang et al. Finally, Wang et al. presented their experience on paraganglioma of the urinary bladder Wang et al. They described 29 patients with a variety of clinical symptoms (hypertension, palpitations and micturition syncope) with some of them showing also increased 24-hour catecholamines and norepinephrine or positive metaiodobenzylguanidine or octreotide scans Wang et al. They also provide insights regarding treatment options and prognosis Wang et al.

Minimally invasive surgical techniques in endourology have been revolutionized by the advent of thulium fiber laser, low cost of treatment, use of suction for fragment removal and focus on patient reported outcome measures (1–4). Similarly, robotic surgery has pioneered new techniques in prostatectomy and partial nephrectomy Leitsmann et al., Katsimperis et al., Chen et al., Nguyen et al., Gu et al. These advances give patients more treatment choices and possibly better outcomes, contributing to personalized patient care. With the advent of artificial intelligence (AI), it is only a matter of time before AI influences all aspects of urological care too (5).
